# MIC Distributions and Evaluation of Fungicidal Activity for Amphotericin B, Itraconazole, Voriconazole, Posaconazole and Caspofungin and 20 Species of Pathogenic Filamentous Fungi Determined Using the CLSI Broth Microdilution Method

**DOI:** 10.3390/jof3020027

**Published:** 2017-05-31

**Authors:** Andrew M. Borman, Mark Fraser, Michael D. Palmer, Adrien Szekely, Marian Houldsworth, Zoe Patterson, Elizabeth M. Johnson

**Affiliations:** Public Health England United Kingdom, Mycology Reference Laboratory, Myrtle Road, Bristol BS2 8EL, UK; mark.fraser@phe.gov.uk (M.F.); michael.palmer@phe.gov.uk (M.D.P); adrien.szekely@phe.gov.uk (A.S.); marian.houldsworth@phe.gov.uk (M.H.); zoe.patterson@phe.gov.uk (Z.P.); elizabeth.johnson@phe.gov.uk (E.M.J.)

**Keywords:** filamentous fungi, susceptibility testing, ECVs, amphotericin B, itraconazole, posaconazole, voriconazole, caspofungin, fungicidal, wild-type distributions

## Abstract

For filamentous fungi (moulds), species-specific interpretive breakpoints and epidemiological cut-off values (ECVs) have only been proposed for a limited number of fungal species–antifungal agent combinations, with the result that clinical breakpoints are lacking for most emerging mould pathogens. In the current study, we have compiled minimum inhibitory concentration (MIC) data for 4869 clinical mould isolates and present full MIC distributions for amphotericin B, itraconazole, voriconazole, posaconazole, and caspofungin with these isolates which comprise 20 species/genera. In addition, we present the results of an assessment of the fungicidal activity of these same five antifungal agents against a panel of 123 mould isolates comprising 16 of the same species.

## 1. Introduction

Infections caused by filamentous fungi (moulds) are increasingly common in immunocompromised hosts, and are associated with high morbidity and mortality [1]. Although *Aspergillus fumigatus* and other *Aspergillus* species remain the most common causal agents [2,3,4], disseminated infections caused by *Fusarium* spp., *Scedosporium* spp., and members of the *Mucoromycotina* (*Rhizopus*, *Lichtheimia*, *Mucor* spp.) and other rarer moulds are associated with particularly poor prognoses [5,6,7,8,9,10,11,12,13]. The emergence of new fungal pathogens has been mirrored by an increasing number of species that are resistant to at least some of the established antifungal agents [14,15,16,17]. As a result of these trends, new antifungal agents continue to find significant clinical use, and the spectrum of antifungal agents continues to grow [18,19].

Given the range of fungal pathogens and the number of available antifungal agents, in vitro antifungal susceptibility testing of individual isolates is recommended for invasive fungal infections in high-risk patients, both in order to optimise treatment strategies and to detect resistant isolates. However, despite the existence of standardised broth microdilution methodologies for the susceptibility testing of filamentous fungi [20,21], species-specific interpretive breakpoints and epidemiological cut-off values (ECVs) have only been proposed for a limited number of fungal species–antifungal agent combinations [22,23,24,25,26,27].

Mainly on the basis of in vitro testing, the activity of the various antifungal agents has been described as fungistatic or fungicidal, depending on the drug, isolate, and test conditions. Amphotericin B has been the most widely used antifungal agent against systemic infections, and exhibits concentration-dependent fungicidal activity against most fungal isolates in vitro [28]. Similarly, the echinocandins inhibit β-1,3-d-glucan synthesis, and are fungicidal against many *Candida* strains [29], but fungistatic against *Aspergillus* spp. Conversely, the azoles—itraconazole and voriconazole—have been described as exhibiting concentration-dependent fungistatic activity against many organisms [28] and fungicidal activity against a selection of others [30,31,32]. At least certain of these results have been confirmed in experimental models of infection in neutropenic rabbits, guinea pigs, and mice (reviewed in [32]).

Today, much uncertainty remains as to the best in vitro indicator of clinical outcome or in vivo susceptibility. For filamentous fungi, with the exception of the minimum inhibitory concentrations (MICs) for *Aspergillus* spp. with the azoles [33], few correlates between MIC and in vivo outcome have been described [34]. A relationship between voriconazole MIC and in vivo efficacy was noted in immunocompromised mice [35], and reasonable in vitro–in vivo correlates do exist for the fluconazole, caspofungin, and amphotericin B minimum fungicidal concentrations (MFCs) obtained with *Candida* spp. and *Aspergillus* spp., at least in animal models of invasive fungal disease (reviewed in [32]). A number of previous studies have attempted to examine the in vitro relationships between MIC and MFC for a limited number of fungal species, each with a subset of antifungal agents, in some cases employing non-standardised methodologies [31,36,37,38]. However, for filamentous fungi, a clear beneficial effect of treatment with fungicidal agents over fungistatic ones on clinical outcome remains unproven [39,40].

Here, we present the MIC distributions for amphotericin B, itraconazole, voriconazole, caspofungin, and posaconazole for 20 species of filamentous fungi (4869 isolates) that were submitted to the UK Mycology Reference Laboratory (MRL) for susceptibility testing by the CLSI broth microdilution method. In addition, we have evaluated the fungicidal activities of those same five antifungals against a subset of 123 fungal isolates representing 16 of these species.

## 2. Materials and Methods

### 2.1. Clinical Isolates for Minimum Inhibitory/Effective Concentration (MIC/MEC) Distribution Analyses

Minimum inhibitory/effective concentration (MIC/MEC) distributions were ascertained for 4869 clinical isolates of filamentous fungi submitted to the MRL for susceptibility testing between 2006–2016. Isolates included *Acremonium* spp. (*n* = 55), *Alternaria* spp. (58), *Aspergillus fumigatus* (2501), *Aspergillus flavus* (372), *Aspergillus niger* (301), *Aspergillus terreus* (115), *Aspergillus versicolor* (28), *Exophiala* spp. (134), *Fusarium* spp. (586), *Lichtheimia corymbifera* (64), *Lomentospora prolificans* (25), *Mucor* spp. (80), *Paecilomyces variotii* (33), *Purpureocillium lilacinum* (42), *Rasamsonia* spp. (29), *Rhizopus arrhizus* (21), *Rhizopus microsporus* (50), *Rhizomucor pusillus* (34), and *Scedosporium apiospermum* species complex (301). Isolates were identified phenotypically according to standard protocols in our laboratory. MICs and MECs were determined according to CLSI guidelines [20] by broth microdilution as described below.

### 2.2. Additional Isolates

For determination of fungicidal activities, the vast majority of mould isolates came from the National Collection of Pathogenic Fungi (NCPF) housed at the MRL, Bristol, although four strains of *Aspergillus terreus* and three strains of *Aspergillus niger* were recent clinical isolates. For moulds, two reference isolates of *A. fumigatus* (NCPF7097 and NCPF7100) were included in all assays as quality controls. The moulds tested comprised 11 isolates of *Aspergillus fumigatus*, 10 isolates each of *Scedosporium apiospermum*, *Lomentospora prolificans*, *Aspergillus flavus*, *Aspergillus terreus*, *Lichtheimia corymbifera*, 9 isolates each of *Aspergillus niger*, *Exophiala dermatitidis*, and *Fusarium solani*, 13 isolates of *Fusarium* spp. (6 isolates of *F. proliferatum*, 4 of *F. oxysporum*, and 3 of *F. verticilloides*), 7 isolates each of *Purpureocillium lilacinum* and *Paecilomyces variotii*, 4 isolates of *Rhizopus arrhizus*, 3 isolates of *Rhizomucor pusillus*, and 1 isolate of *Rhizopus microsporus*. NCPF isolates were retrieved from storage in liquid nitrogen or water, subcultured on plates of Oxoid Sabouraud dextrose agar (Unipath Ltd., Basingstoke, UK) supplemented with 0.5% (*w*/*v*) chloramphenicol, and incubated at 30 °C. Mould strains were subcultured onto slopes of Oxoid potato dextrose agar and incubated at 35 °C for 7 days to induce sporulation, prior to testing.

### 2.3. Antifungal Agents and Drug Concentration Ranges

Antifungal drugs were obtained from their respective manufacturers as standard powders. Amphotericin B (Sigma Chemical Co., St. Louis, MO, USA) and voriconazole (Pfizer Central Research, Sandwich, UK) were dissolved in dimethyl sulfoxide. Itraconazole (Janssen Research Foundation, Beerse, Belgium) and posaconazole (Merck, Sharp and Dohme, Hoddesdon, UK) were dissolved in PEG400 by heating to 70 °C. Caspofungin (Merck, Sharp and Dohme) was resuspended in sterile water. Serial two-fold dilutions of the various drugs were prepared in RPMI 1640 medium (with l-glutamine, without bicarbonate; Sigma Chemical Co.) and buffered to pH 7.0 using a 0.165 M solution of MOPS (Sigma Chemical Co.). The antifungal agents were tested over a range of final concentrations (0.03 to 16 µg/mL for amphotericin B, voriconazole, posaconazole, and itraconazole; 0.125 to 64 µg/mL for caspofungin).

### 2.4. CLSI Broth Microdilution Determination of Mould MIC and MEC

MICs were determined in round-bottomed 96-well plates with mould conidial suspensions prepared in RPMI 1640 and adjusted to a final concentration of (0.4–5) × 10^4^ CFU/mL as previously described [20]. Inoculated plates were incubated for 48 h at 35 °C except for *L. corymbifera*, *R. arrhizus*, *R. pusillus*, and *R. microsporus*, which were incubated for 24 h. MICs were read at 24 or 48 h as the concentration of drug that elicited 100% inhibition of growth (amphotericin B, itraconazole, posaconazole, voriconazole) or as the minimum effective concentration (MEC, caspofungin), in which the end-point is read as the lowest concentration at which the fungal hyphae can be seen to be stunted with swollen tips.

### 2.5. Determination of Minimum Fungicidal Concentrations

MFCs were determined after 48 h incubation (except for *L. corymbifera*, *R. arrhizus*, *R. pusillus*, and *R. microsporus*, which were determined at 24 h) by removing 10 µL of the contents from wells showing no visible growth and spreading them on to Sabouraud dextrose agar plates. The plates were then incubated for 72 h and MFCs were determined as the lowest drug concentrations which killed 95% of the inoculum.

### 2.6. Data Analysis

MIC and MFC ranges and the drug concentrations required to inhibit or kill 50% (MIC_50_ and MFC_50_, respectively) or 90% of isolates (MIC_90_ and MFC_90_, respectively) were determined for all species that comprised at least seven isolates. For species comprising less than seven isolates, only MIC and MFC ranges were determined.

## 3. Results

The results of in vitro susceptibility testing of 4689 clinical isolates of filamentous fungi submitted to the MRL are summarised in Table 1, Table 2, Table 3, Table 4 and Table 5. In all tests included in the data analysis, the MICs of the control reference strains were within the accepted limits (data not shown). CLSI wild-type MIC distributions and ECVs have only been proposed for *Aspergillus* spp. [23,25,26,27], and some members of the Mucoromycotina [22]. For the more commonly encountered *Fusarium* spp., ECVs have been proposed using CLSI methodology [41], but have not been formally accepted by CLSI. In the current study, we could not establish wild-type distributions or ECVs for the various organisms studied, as under CLSI guidelines they can only be defined when more than 100 MIC results per species are available from at least three independent laboratories.

MIC data for amphotericin B were broadly concordant with previously published international studies [22,23,41]. Organisms for which significant proportions of isolates had MICs in excess of the ECV for *A. fumigatus* included *A. terreus* and *Fusarium* spp., for which higher ECVs have been proposed [23,41] and *Lomentospora prolificans*, *Purpureocillium lilacinum*, *Rasamsonia* spp. and *Scedosporium apiospermum* species complex. Previous studies have highlighted elevated amphotericin B MICs with these organisms [42,43,44], and amphotericin B is not currently suggested as optimal first line treatment for those organisms [45]. For the Mucoromycotina, MIC ranges were predominantly below the ECV of *A. fumigatus*, in agreement with previous studies that specifically aimed to define ECVs for these organisms [22].

With itraconazole, again most isolates of many of the species examined had MICs below the ECV proposed for *A. fumigatus* (Table 2). Organisms with heavily skewed MIC distributions included *Acremonium* spp., *Fusarium* spp., and *Lo. prolificans*. Once again, current treatment guidelines do not recommend itraconazole for infections by these organisms [45]. In agreement with previous studies involving the Mucoromycotina [22], the MICs for *Mucor* spp., *Rhizopus* spp., and *Rhizomucor pusillus* were considerably higher than those observed with *L. corymbifera*, with concomitantly higher ECVs having been proposed for the former organisms [22]. It should be noted that approximately 5% of isolates of *A. fumigatus* from the current study had MICs above the proposed ECV of 1 mg/L with itraconazole. This is likely to result from patient bias in the samples received by the MRL, where a high proportion of isolates are from patients with cystic fibrosis and are thus likely to have had previous itraconazole exposure (data not shown).

Posaconazole MIC distributions are given in Table 3. MIC ranges significantly higher than the ECV for *A. fumigatus* were observed with *Fusarium* spp., *Lo. prolificans*, and certain members of the Mucoromycotina, in broad agreement with previous reports [8,22,41]. Of the antifungal agents studied here, amphotericin B and posaconazole appear to exert the most activity against the Mucoromycotina in vitro, in agreement with previous studies and current treatment recommendations [8].

Depending on infection site, voriconazole is recommended as first-line treatment for infections with *S. apiospermum*, *Aspergillus* spp., *Acremonium* spp., *Fusarium* spp., and *Lo. prolificans* (in combination with terbinafine) [45]. For *Acremonium* spp., *S. apiospermum*, and all of the *Aspergillus* spp. examined here, MIC values predominantly fell below the ECV proposed for *A. fumigatus* [26] (Table 4). For *Fusarium* spp., MICs were lower with voriconazole than with either itraconazole or posaconazole, and clinical treatment successes have been observed with voriconazole—especially when accompanied by surgical interventions and immune reconstitution [45]. Similarly, voriconazole is superior to itraconazole and posaconazole in vitro towards *Lo. prolificans*, and has been associated with 40–50% survival rates especially when used in conjunction with terbinafine [45]. Voriconazole had little apparent in vitro activity against the remaining species examined here (members of the Mucoromycotina, *P. variotii*, *Rasamsonia* spp.), with MIC ranges significantly higher than the ECV for *A. fumigatus*.

The MEC ranges observed with caspofungin were significantly higher than the proposed ECV for *A. fumigatus* for all organisms except *Aspergillus* spp. and *Rasamsonia* spp. (Table 5). For *A. nidulans* and to a lesser extent *A. versicolor*, MEC ranges appeared to be bimodal as described previously [23], with a small proportion of isolates having MECs significantly above the *A. fumigatus* ECV. The apparent lack of in vitro activity of caspofungin against *Fusarium* spp., members of the Mucoromycotina, *S. apiospermum*, *Lo. prolificans*, *P. variotii*, *P. lilacinum*, and *Exophiala* spp. has been reported previously [46], and correlates well with the lack of in vivo success of the echinocandin antifungal agents as primary treatment of infections with these various organisms, which presumably is a result of the reduced concentrations of 1,3-β-d-glucan in the cell walls of many of these species [47,48].

Certain studies have suggested that in vivo outcome may be improved when fungicidal as opposed to fungistatic agents are employed for treatment, although the evidence with invasive mould infections is less than compelling [39]. To evaluate potential species-specific differences in the fungicidal versus fungistatic actions of the five antifungal agents included in the current study, a subset of 123 isolates from the NCPF were subjected to susceptibility testing by CLSI broth microdilution followed by end-point plating to determine MFCs (see Section 2). The ranges of MICs for the various drug–organism combinations were indistinguishable from those observed in our analyses of the 4869 clinical isolates referred to the MRL from various UK hospital centre microbiology laboratories (data not shown).

The MFC ranges for the five different drugs were, however, significantly different for the various organisms (Table 6). Amphotericin B exhibited fungicidal activity against isolates of *A. fumigatus*, *A. flavus*, *A. niger*, *E. dermatitidis*, *L. corymbifera*, *R. arrhizus*, and *R. microsporus* (MFC_50_s where appropriate <1 µg/mL). Indeed, the MFCs of amphotericin B for these organisms rarely exceeded the modal MICs observed in our larger survey by more than 1–2 doubling dilutions (Table 6). Itraconazole, posaconazole, and voriconazole also exhibited significant fungicidal activity, killing at least 50% of isolates of *A. fumigatus*, *A. flavus*, *A. niger*, *A. terreus*, and *E. dermatitidis.* Voriconazole and posaconazole were the only antifungal drugs tested to demonstrate any fungicidal activity against isolates of *S. apiospermum* or *P. lilacinum*, whereas the same was true for itraconazole and posaconazole with isolates of *P. variotii*. Posaconazole was the only triazole antifungal agent tested to exhibit at least limited fungicidal activity against some members of the Mucoromycotina. No significant fungicidal activity could be detected for any mould-drug combination with caspofungin.

## 4. Discussion

The aim of antifungal susceptibility testing is to obtain a result than can be used to determine the likelihood of treatment success of an infection by a particular organism with the antifungal agent in question. Ideally, this interpretive decision relies upon the development of clinical breakpoints (CBPs) that are intended to predict therapeutic outcome. With yeast isolates, there is a suggestion that a “90:60 rule” should be applied, which proposes that susceptible organisms will respond to therapy 90% of the time whilst resistant organisms would only respond in 60% of cases [49]. As most patients with invasive mould infection are immunocompromised, the lack of adjunctive host response is likely to have an additional impact on the probable outcome of therapy. Indeed, in most clinical trials of invasive aspergillosis, there appears to be an irreducible 40% mortality rate even with susceptible organisms [50], and there is a wealth of anecdotal evidence and case histories to support the contention that mould isolates that are resistant in vitro will rarely—if ever—respond to therapy. Therefore, for moulds, perhaps the rule needs revision to a “10:60 rule” in which susceptible organisms will respond in 60% of cases and resistant organisms will only respond in 10%.

Unfortunately, the development of CBPs is currently impossible for many filamentous fungal (mould) infections, principally due to the lack of sufficient clinical trial data for some of the rarer, emerging pathogens. In the absence of CBPs, ECVs can be used to determine whether a particular isolate may have acquired resistance to a particular antifungal agent via the identification of isolates with “non-wild-type” MICs. The current study was not geared towards developing ECVs, since they require the analysis of at least 100 independent isolates of a particular species, with MIC values obtained from at least three independent centres. However, the current study is intended to contribute to the existing literature and aid the future development of ECVs and CBPs via the analysis of the antifungal susceptibility profiles of a large number of clinical isolates from across the UK. Moreover, in the absence of specific antifungal susceptibility testing on the causative organism, the current data may help clinicians dealing with infections with rarer moulds to decide which antifungal drug may be most likely to be effective. The MIC distributions reported here for *Aspergillus* spp., *Fusarium* spp., and members of the Mucoromycotina are largely similar to those previously reported from large international studies involving the USA [23,25,26,27,41], suggesting that UK isolates are broadly similar to their US counterparts. Additional studies have suggested species-specific antifungal profiles and individual ECVs for different *Fusarium* spp. and for individual cryptic species within the *S. apiospermum* species complex [41,42]. Unfortunately, it was beyond the remit of the current study to identify individual *Fusarium* and *S. apiospermum* species to cryptic species level, and MIC data were grouped for the whole series in each case. Nevertheless, the MIC distributions reported here are very similar to those reported previously with individual species [41,42].

In the absence of ECV and CBP data, MIC distributions may still be clinically useful in identifying bimodal distributions indicative of sub-populations with acquired or intrinsic resistance. Similarly, identification of antifungal agent–organism combinations where MIC ranges are always elevated compared to other species may aid in eliminating particular therapeutic approaches. A number of such combinations including both yeast and mould species have become evident over the last decades, and will not be discussed in detail here. Examples include members of the Mucoromycotina, *Scedosporium* spp., *Lo. prolificans*, and *Fusarium* spp. with the echinocandin antifungals, and *A. terreus* with amphotericin B. From the current study, and in the absence of contradictory clinical data suggesting therapeutic benefits, such combinations may be extended to include *Acremonium* (*Sarocladium*) and *Rasamsonia* spp. with amphotericin B, *Acremonium* (*Sarocladium*) spp. with itraconazole, *Rasamsonia* spp., *Alternaria* spp., and *P. variotii* with voriconazole, *Acremonium* spp. with posaconazole, and *Acremonium* spp., *Exophiala* spp., *P. variotii*, and *P. lilacinum* with caspofungin. Some clinical support exists to suggest that these combinations might not be clinically indicated. For instance, *Rasamsonia* spp. have been reported as the cause of disseminated infections in CGD patients receiving long-term triazole prophylaxis [51]; several case reports exist of clinical failures with disseminated infections due to *Acremonium* spp. treated with amphotericin B and itraconazole [52,53,54]; amphotericin B was ineffective in disseminated human infections caused by *P. lilacinum* [55], and did not reduce tissue burden in a murine model of disseminated *P. lilacinum* infection [56]; breakthrough infections and treatment failures have been reported with voriconazole and *P. variotii* [57,58]. Similarly, for *Alternaria* spp., we have increasing anecdotal evidence of clinical failures when voriconazole was employed to treat subcutaneous infections (MRL unpublished data).

In the current study, we have also evaluated which of the five test antifungals exert fungicidal effects on common and emerging filamentous fungal pathogens, using a subset of isolates from the NCPF that had been reliably identified using molecular approaches. The amphotericin B, itraconazole, and voriconazole MFC values reported here for *Aspergillus* spp., *E. dermatitidis*, *S. apiospermum*, *R. arrhizus*, and *L. corymbifera* are in very good agreement with a previous study conducted by us some eight years ago, using different test isolates [31]. Overall, the data confirm the fungicidal activities of amphotericin B, itraconazole, and voriconazole for isolates of *Exophiala dermatitidis* and for *Aspergillus* spp. (with the exception of the well-documented resistance to amphotericin B for isolates of *A. terreus*; Table 6; [38]). Coupled with the lack of demonstrable fungicidal activity of caspofungin against any of the filamentous fungi tested here, these findings also agree extremely well with in vivo neutropenic animal models of invasive aspergillosis [59,60].

## 5. Conclusions

In summary, here we have presented MIC distributions for 4869 clinical isolates of filamentous moulds corresponding to 20 different genera/species. For those organisms that had been previously studied elsewhere, our MIC ranges were in very good agreement with previous reports. The additional MIC ranges presented here for rarer, emerging pathogens (*Acremonium* spp., *Alternaria* spp., *Exophiala* spp., *Rasamsonia* spp., *P. variotii*, *P. lilacinum*, *Lo. prolificans*) are intended to add to the existing body of literature with the aim of facilitating the development of ECVs for these organisms and the detection of isolates with non-wildtype MICs. However, as ECVs do not predict the clinical outcome of therapy (an isolate with a susceptible or wild-type MIC might not necessarily respond in vivo to that particular antifungal agent), further studies will be required to ascertain the molecular basis for resistance in these particular rarer pathogens in order to establish robust CBPs.

## Figures and Tables

**Table 1:** Minimum inhibitory concentration (MIC) distributions for amphotericin B and common pathogenic filamentous fungi. The number of isolates included is given in parentheses, together with the number of isolates with each given MIC value. %R denotes the proportion of isolates with non-wild type MICs (i.e., MICs greater than the CLSI 97.5% epidemiological cutoff value (ECV) proposed for *A. fumigatus*, dashed line [23],) with percentages above 30% in bold face. Dashed Line: no isolates with this MIC.

Antifungal/Organism	MIC (mg/L)										
	0.03	0.06	0.125	0.25	0.5	1	2	4	8	16	%R
Amphotericin B											
*Acremonium* sp. (55)	-	1	-	2	5	9	15	15	3	5	**69.1**
*Alternaria* sp. (58)	-	3	7	24	20	4	-	-	-	-	0
*Aspergillus flavus* (372)	-	-	2	16	104	199	42	9	-	-	13.7
*Aspergillus fumigatus* (2501)	-	1	11	134	778	1265	310	2	-	-	12.5
*Aspergillus nidulans* (40)	2	1	2	8	13	12	2	-	-	-	5
*Aspergillus niger* (301)	6	49	117	101	27	1	-	-	-	-	0
*Aspergillus terreus* (115)	-	-	-	2	16	42	43	7	5	-	**47.8**
*Aspergillus versicolor* (28)	-	-	-	2	9	10	7	-	-	-	25
*Exophiala* sp. (134)	1	2	14	33	67	17	-	-	-	-	0
*Fusarium* sp. (586)	-	1	3	6	71	304	153	40	8	-	**34.3**
*Lichtheimia corymbifera* (64)	1	5	13	28	13	4	-	-	-	-	0
*Lomentospora prolificans* (25)	-	-	-	-	-	-	5	8	5	7	**100**
*Mucor* sp. (80)	5	9	22	21	19	3	-	-	1	-	1.2
*Paecilomyces variotii* (33)	3	10	5	6	3	4	2	-	-	-	6.1
*Purpureocillium lilacinum* (42)	-	-	-	-	-	-	1	-	1	40	**100**
*Rasamsonia* sp. (29)	-	-	1	-	5	8	6	6	3	-	**31**
*Rhizopus arrhizus* (21)	-	1	5	6	7	2	-	-	-	-	0
*Rhizopus microsporus* (50)	-	1	6	19	20	3	1	-	-	-	2
*Rhizomucor pusillus* (34)	1	1	7	18	7	-	-	-	-	-	0
*Scedosporium apiospermum* (301)	-	1	3	10	34	94	65	58	14	22	**52.8**

**Table 2:** MIC distributions for Iitraconazole. For presentation conventions, see Legend to Table 1.

Antifungal/Organism	MIC (mg/L)											
	0.03	0.06	0.125	0.25	0.5	1	2	4	8	16	32	%R
Itraconazole												
*Acremonium* sp. (50)	-	1	1	3	3	7	2	3	3	-	27	**70**
*Alternaria* sp. (68)	3	7	20	28	8	1	1	-	-	-	-	1.5
*Aspergillus flavus* (244)	34	91	82	29	8	-	-	-	-	-	-	0
*Aspergillus fumigatus* (2268)	22	453	629	632	370	53	24	21	9	45	10	4.8
*Aspergillus nidulans* (39)	7	9	14	9	-	-	-	-	-	-	-	0
*Aspergillus niger* (149)	1	6	40	48	51	10	3	-	-	-	-	2
*Aspergillus terreus* (98)	23	41	25	5	4	-	-	-	-	-	-	0
*Aspergillus versicolor* (21)	2	1	4	6	6	1	1	-	-	-	-	4.7
*Exophiala* sp. (139)	7	12	34	51	26	7	2	-	-	-	-	1.4
*Fusarium* sp. (488)	-	-	-	4	18	16	26	18	2	404	-	**98.2**
*Lichtheimia corymbifera* (56)	2	2	13	24	14	1	-	-	-	-	-	0
*Lomentospora prolificans* (25)	-	-	-	-	-	-	-	-	-	25	-	**100**
*Mucor* sp. (76)	2	2	1	4	12	15	7	1	-	32	-	**52.6**
*Paecilomyces variotii* (30)	11	7	2	3	1	1	2	1	2	-	-	16.7
*Purpureocillium lilacinum* (38)	1	-	-	3	20	11	-	2	-	2	-	10.5
*Rasamsonia* sp. (30)	1	-	2	2	10	8	4	2	-	1	-	23.3
*Rhizopus arrhizus* (19)	-	-	-	1	4	5	3	1	1	4	-	**47.4**
*Rhizopus microsporus* (46)	-	-	-	4	16	15	7	-	-	4	-	23.9
*Rhizomucor pusillus* (29)	2	4	-	7	7	3	3	-	-	3	-	20.7
*Scedosporium apiospermum* (287)	3	7	23	89	111	29	7	8	2	8	-	8.7

**Table 3:** MIC distributions for posaconazole. For presentation conventions, see Legend to Table 1.

Antifungal/organism	MIC (mg/L)										
	0.03	0.06	0.125	0.25	0.5	1	2	4	8	16	%R
Posaconazole											
*Acremonium* sp. (8)	-	1	2	1	1	3	-	-	-	-	**37.5**
*Alternaria* sp. (32)	7	18	6	1	-	-	-	-	-	-	0
*Aspergillus flavus* (83)	21	44	15	3	-	-	-	-	-	-	0
*Aspergillus fumigatus* (396)	111	150	65	37	21	8	2	-	1	1	3
*Aspergillus nidulans* (7)	1	4	1	1	-	-	-	-	-	-	0
*Aspergillus niger* (43)	3	25	10	4	1	-	-	-	-	-	0
*Aspergillus terreus* (37)	18	15	3	-	-	-	1	-	-	-	2.7
*Aspergillus versicolor* (7)	1	1	1	2	2	-	-	-	-	-	0
*Exophiala* sp. (34)	3	11	12	4	4	-	-	-	-	-	0
*Fusarium* sp. (124)	-	-	3	6	19	19	18	5	1	53	**77.4**
*Lichtheimia corymbifera* (58)	-	3	16	26	13	-	-	-	-	-	0
*Lomentospora prolificans* (21)	-	-	-	-	1	1	3	-	16	-	**95.2**
*Mucor* sp. (79)	3	3	3	9	18	21	10	6	1	5	**41.8**
*Paecilomyces variotii* (19)	9	5	1	1	2	-	1	-	-	-	5.5
*Purpureocillium lilacinum* (13)	1	4	2	5	-	1	-	-	-	-	7.7
*Rasamsonia* sp. (13)	1	-	2	-	8	2	-	-	-	-	15.4
*Rhizopus arrhizus* (20)	-	-	2	3	6	7	-	-	-	2	**45**
*Rhizopus microsporus* (49)	-	1	1	13	27	1	3	2	-	1	14.3
*Rhizomucor pusillus* (32)	-	7	2	5	12	4	1	1	-	-	18.7
*Scedosporium apiospermum* (85)	2	2	10	39	26	2	1	1	-	2	7

**Table 4:** MIC distributions for voriconazole. For presentation conventions, see Legend to Table 1.

Antifungal/organism	MIC (mg/L)										
	0.03	0.06	0.125	0.25	0.5	1	2	4	8	16	%R
Voriconazole											
*Acremonium* sp. (55)	-	2	5	11	20	11	3	1	1	1	10.9
*Alternaria* sp. (63)	-	-	7	6	13	20	6	8	3	1	28.6
*Aspergillus flavus* (283)	-	5	72	154	47	3	1	1	-	-	0.7
*Aspergillus fumigatus* (2384)	1	13	309	1631	299	66	45	13	-	7	3.1
*Aspergillus nidulans* (39)	2	14	19	2	2	-	-	-	-	-	0
*Aspergillus niger* (164)	2	3	24	82	47	6	-	-	-	-	0
*Aspergillus terreus* (106)	-	1	31	55	16	1	2	-	-	-	1.9
*Aspergillus versicolor* (22)	-	3	7	5	6	-	1	-	-	-	4.5
*Exophiala* sp. (126)	23	20	42	25	14	8	11	1	2	-	11.1
*Fusarium* sp. (593)	2	2	11	11	46	151	185	153	31	1	**62.4**
*Lichtheimia corymbifera* (50)	-	-	-	-	-	-	7	12	16	15	**100**
*Lomentospora prolificans* (25)	-	-	-	-	-	9	10	6	-	-	**64**
*Mucor* sp. (73)	-	-	2	-	-	-	1	3	4	63	**97.3**
*Paecilomyces variotii* (30)	-	-	-	2	2	1	10	2	1	12	**83.3**
*Purpureocillium lilacinum* (43)	2	2	22	13	3	-	-	-	1	-	2.3
*Rasamsonia* sp. (30)	-	-	-	-	-	-	-	3	-	27	**100**
*Rhizopus arrhizus* (18)	-	-	-	-	-	-	2	3	6	7	**100**
*Rhizopus microsporus* (36)	-	-	-	-	-	-	1	15	17	2	**100**
*Rhizomucor pusillus* (30)	-	-	1	-	-	3	1	3	5	17	**86.7**
*Scedosporium apiospermum* (304)	9	51	101	114	19	1	7	1	1	-	2.9

**Table 5:** Minimum effective concentration (MEC) distributions for caspofungin. For presentation conventions, see Legend to Table 1.

Antifungal/Organism	MEC (mg/L)												
	0.015	0.03	0.06	0.125	0.25	0.5	1	2	4	8	16	32	%R
Caspofungin													
*Acremonium* sp. (11)	-	-	-	-	1	1	1	3	1	-	1	3	**72.7**
*Alternaria* sp. (20)	-	-	-	1	5	5	3	1	-	-	1	3	25
*Aspergillus flavus* (134)	15	12	6	14	74	5	3	4	-	-	-	1	3.7
*Aspergillus fumigatus* (946)	2	64	100	86	594	89	-	-	1	2	4	4	1.2
*Aspergillus nidulans* (18)	-	-	2	2	6	2	1	4	1	-	-	-	27.8
*Aspergillus niger* (83)	-	13	7	9	48	6	-	-	-	-	-	-	0
*Aspergillus terreus* (43)	-	2	5	4	32	-	-	-	-	-	-	-	0
*Aspergillus versicolor* (17)	-	2	1	1	5	5	-	1	2	-	-	-	17.6
*Exophiala* sp. (33)	1	-	-	-	1	-	1	7	2	4	1	16	**90.9**
*Fusarium* sp. (81)	-	-	-	-	2	1	-	2	5	9	12	50	**96.3**
*Lichtheimia corymbifera* (10)	-	-	-	-	-	-	-	-	-	1	-	9	**100**
*Lomentospora prolificans* (10)	-	-	-	-	-	-	-	-	2	3	4	1	**100**
*Mucor* sp. (13)	-	-	-	-	-	-	-	-	-	-	-	13	**100**
*Paecilomyces variotii* (16)	-	-	1	1	4	3	-	5	1	-	1	-	**43.7**
*Purpureocillium lilacinum* (6)	-	-	-	1	-	1	-	2	1	-	-	1	**66.7**
*Rasamsonia* sp. (5)	1	1	1	2	-	-	-	-	-	-	-	-	0
*Rhizopus arrhizus* (7)	-	-	-	-	-	-	-	-	-	-	2	5	**100**
*Rhizopus microsporus* (10)	-	-	-	-	-	-	-	-	-	1	-	9	**100**
*Rhizomucor pusillus* (5)	-	-	-	-	-	-	-	-	-	-	-	5	**100**
*Scedosporium apiospermum* (98)	-	-	2	2	4	4	20	33	4	10	3	13	**64.3**

**Table 6:** Minimum fungicidal concentration (MFC) data for mould isolates. Fungicidal activities are highlighted in grey (i.e., fungicidal effect observed at or below ECV MIC for *A. fumigatus* with that antifungal agent). na—Not appropriate due to the small number of isolates tested for the species. nd—Not done. * *Fusarium* spp. included *F. proliferatum* (6), *F. verticillioides* (3), and *F. oxysporum* (4) species complexes.

Species (No. Tested)	Antifungal Agent	MFC (mg/mL)		
		Range	MFC_50_	MFC_90_
*S. apiospermum*	Amphotericin B	0.6–>16	2	>16
(10)	Itraconazole	0.5–>16	16	>16
	Voriconazole	0.25–>16	1	>16
	Caspofungin	4–>64	32	>64
	Posaconazole	<0.03–0.5	0.5	0.5
*Lo. prolificans*	Amphotericin B	4–>16	8	>16
(10)	Itraconazole	>16	>16	>16
	Voriconazole	>16	>16	>16
	Caspofungin	16–>64	32	>64
	Posaconazole	>16	>16	>16
*F. solani*	Amphotericin B	0.25–>16	2	4
(9)	Itraconazole	>16	>16	>16
	Voriconazole	8–>16	16	>16
	Caspofungin	64–>64	>64	>64
	Posaconazole	1–>16	>16	>16
*Fusarium* spp. *	Amphotericin B	1–4	2	4
(13)	Itraconazole	>16	>16	>16
	Voriconazole	4–>16	16	16
	Caspofungin	>64	>64	>64
	Posaconazole	1–>16	2	>16
*A. fumigatus*	Amphotericin B	0.5–2	1	2
(11)	Itraconazole	0.25–>16	0.5	2
	Voriconazole	0.25–8	1	4
	Caspofungin	64–>64	>64	>64
	Posaconazole	<0.03–0.25	<0.03	0.125
*A. flavus*	Amphotericin B	1–2	1	2
(10)	Itraconazole	0.06–0.25	0.125	0.25
	Voriconazole	0.25–8	0.5	1
	Caspofungin	8–>64	64	>64
	Posaconazole	0.06	0.06	0.06
*A. niger*	Amphotericin B	0.25–2	0.5	2
(9)	Itraconazole	0.25–0.5	0.25	0.5
	Voriconazole	0.25–1	0.5	1
	Caspofungin	32–64	32	64
	Posaconazole	<0.03–0.125	0.06	0.125
*A. terreus*	Amphotericin B	8–>16	>16	>16
(10)	Itraconazole	0.25–4	0.5	4
	Voriconazole	0.5–>16	2	>16
	Caspofungin	32–>64	>64	>64
	Posaconazole	<0.03–1	0.125	0.5
*E. dermatitidis*	Amphotericin B	0.5–2	0.5	2
(9)	Itraconazole	0.125–0.5	0.25	0.5
	Voriconazole	0.25–>16	0.5	>16
	Caspofungin	8–>64	32	>64
	Posaconazole	nd		
*P. variotii*	Amphotericin B	1–>16	16	>16
(7)	Itraconazole	0.25–16	0.5	16
	Voriconazole	4–>16	>16	>16
	Caspofungin	16–64	16	64
	Posaconazole	0.125–16	0.25	16
*P. lilacinum*	Amphotericin B	4–>16	8	>16
(7)	Itraconazole	0.5–>16	>16	>16
	Voriconazole	0.25–2	1	2
	Caspofungin	>64	>64	>64
	Posaconazole (4)	0.06–0.5	na	na
*L. corymbifera*	Amphotericin B	0.5–>16	1	4
(10)	Itraconazole	1–>16	4	>16
	Voriconazole	>16	>16	>16
	Caspofungin	>64	>64	>64
	Posaconazole	0.06–>16	0.25	>16
*Rhizopus* spp.	Amphotericin B	0.25–1	na	na
(5)	Itraconazole	0.25–>16	na	na
	Voriconazole	8–>16	na	na
	Caspofungin	>64	>64	>64
	Posaconazole	0.125>16	na	na
*R. pusillus*	Amphotericin B	0.5–16	na	na
(3)	Itraconazole	1–>16	na	na
	Voriconazole	>16	na	na
	Caspofungin	>64	>64	>64
	Posaconazole	>16	>16	>16
